# Current understanding of heparanase 2 regulation, a non-heparanase

**DOI:** 10.1042/BST20241281

**Published:** 2025-02-05

**Authors:** Yannic Becker, Hermann Haller

**Affiliations:** 1 Department of Nephrology, Hannover Medical School, Hannover, Germany; 2 Mount Desert Island Biological Laboratory MDIBL, Bar Harbor, Maine, USA

**Keywords:** heparan sulfate, heparanase, heparanase 2, proteoglycan, urofacial syndrome

## Abstract

Heparan sulfate (HS) proteoglycans are life-supporting proteins comprising a core protein to which one or more HS glycan chains are covalently bound. HS proteoglycans act as binding sites for circulating cells and molecules, allow gradient formation, and provide local storage capacities. They act as coreceptors, fine-tuning growth factor receptors and activating intracellular signaling pathways. HS glycan chains are cleaved and regulated by heparanase 1 (Hpa1). Heparanase 2 (Hpa2) is a close homolog of Hpa1. Unlike Hpa1, Hpa2 lacks enzymatic activity but nonetheless binds HS with high affinity, thus modulating HS-mediated biological processes. Only a few functions of Hpa2 have been unraveled. Under disease conditions that include the Mendelian urofacial syndrome, Hpa2 expression is markedly down-regulated, most compellingly demonstrated in several cancers. Hpa2 also circulates in the bloodstream, potentially originating from secretory organs such as liver and pancreas. The *Hpa2* promotor is inducible by cellular stressors including cytotoxic, proteostatic, and endoplasmic reticulum stress. Activating transcription factor 3 (ATF3) induces *Hpa2* gene expression. We summarize Hpa2 regulation in the framework of health and disease to foster research into its function. The underlying mystery remains: ‘How does this “heparanase,” which is actually a non-heparanase, work, and what are the ramifications?

## Heparanase 2—a high-affinity heparan sulfate-binding protein

In 2000, McKenzie and colleagues cloned a human gene that shares significant sequence homology with endoglycosidase heparanase 1 (*Hpa1*); thus, they termed it heparanase 2 (*Hpa2*) [1]. Subsequent biochemical analyses, however, revealed that Hpa2 is in fact not a heparanase, as it possesses no enzymatic activity toward heparin or heparan sulfate (HS) [2], and thus its name—designating it an enzyme—is misleading. Nevertheless, Hpa2 has even greater affinity for HS than does Hpa1, which established the thought model that Hpa2 can block Hpa1 activity via competitive inhibition [2].

HS belongs to the glycosaminoglycans (GAGs), long linear polysaccharides consisting of repeating disaccharide units. GAGs are classified based on their disaccharide structures. HS chains are made of two alternating monosaccharides: N-acetylglucosamine and hexuronic acid. HS undergoes various chain modifications including deacetylation, epimerization, and sulfation during the maturation process in the Golgi compartment [3]. GAGs are attached to core proteins, and the resulting glycoprotein is called proteoglycan. Proteoglycans that include HS are essential for normal cell physiology because of their strategic localization in basement membranes, at the cell surface, and in intracellular granules. Among others, they are important for cell–matrix interactions, act as coreceptors, create gradients for morphogens, and protect proteins from proteolytic degradation. Therefore, HS is indispensable to vertebrate life and, concordantly, is also associated with multiple diseases [4,5].

**Figure 1:**
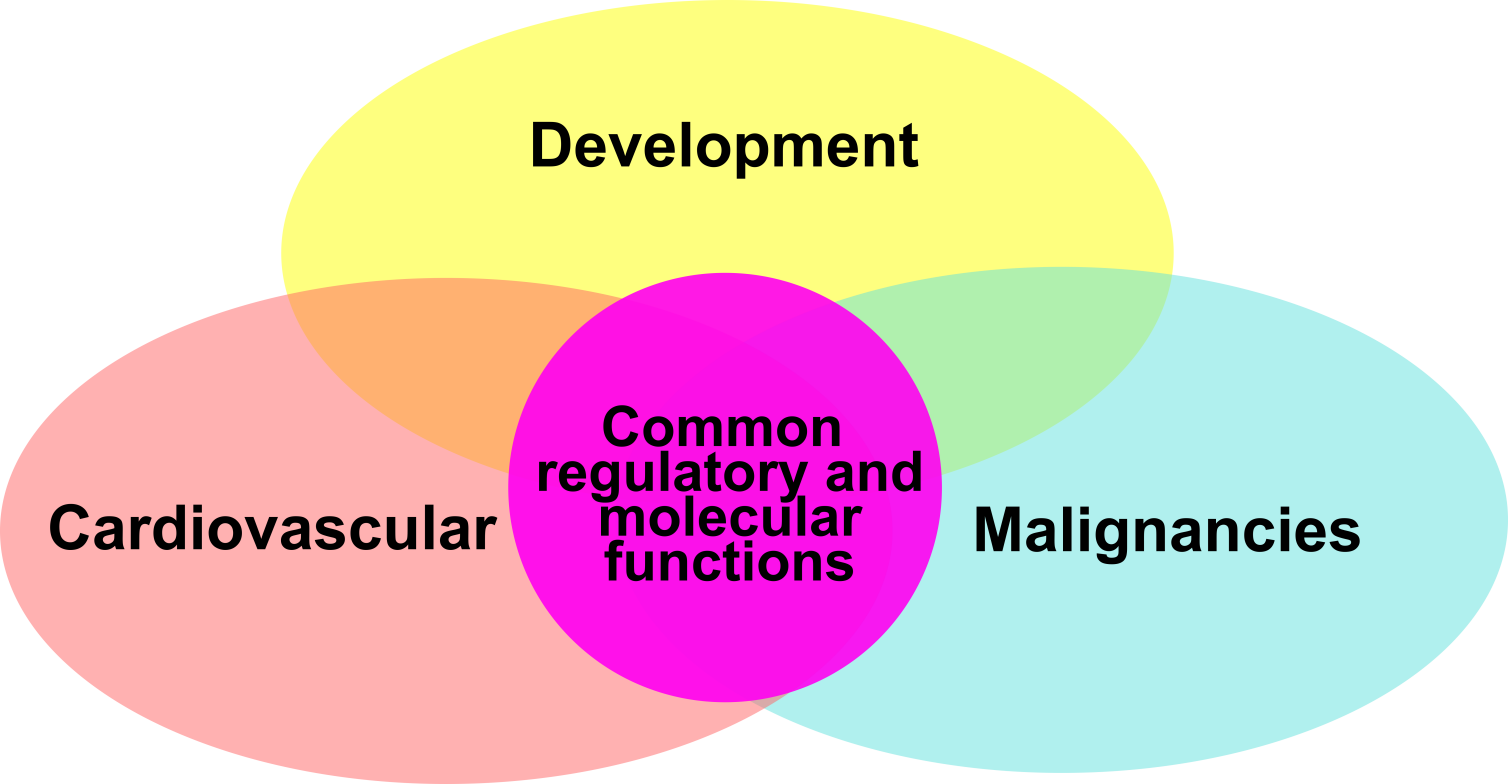
The roles of Hpa2 in different biological areas. Hpa2 plays a role in vertebrate peripheral nervous system development, the cardiovascular system, and malignant transformation. Common regulatory mechanisms remain to be investigated, and the different areas linked by understanding the molecular functions of Hpa2.

Hpa2 competes with Hpa1 for binding HS. Other HS-binding proteins may compete in a similar manner with Hpa2 for HS binding. We recently showed that Hpa2 competes with HS-dependent growth factors fibroblast growth factor 2 and vascular endothelial growth factor A_165_ on endothelial cells for HS binding [6]. This suggests a broader role for Hpa2 in regulating HS-dependent processes. However, the physiological consequences of the Hpa2–HS interaction remain incompletely understood. Research over the past two decades has identified several physiological functions for Hpa2 across different organs and developmental stages (Figure 1). The most comprehensively studied function of Hpa2 is its role in peripheral nervous system development. Humans with biallelic mutations (autosomal-recessive) in *Hpa2* develop urofacial syndrome (UFS), a disease characterized by dyssynergia of bladder innervation and abnormal facial expression [7–9]. The neuronal defects of bladder innervation are mirrored in Hpa2-deficient mice, which also exhibit growth retardation and die within a month after birth [10,11]. In addition, Hpa2 expression was shown to be down-regulated in multiple malignant tissues including head and neck [2,12], gastric [13,14], breast [15], bladder [16], colon [17], and pancreatic carcinomas [18], strongly suggesting that Hpa2 could have a tumor-suppressor function. Accordingly, xenograft models generated by implanting Hpa2-overexpressing cells into immunodeficient mice confirmed the tumor-inhibiting effect of Hpa2 [12,14,16,18]. Investigators have also found that the Hpa2 protein is prominent in the vasculature [19–21], and that Hpa2 plays a necessary role in maintaining vascular integrity by regulating HS–growth factor interactions on the vascular surface [6]. Together, these findings suggest that Hpa2 functions in a manner opposite to Hpa1, which is well known for its HS-degrading activity, thereby acting as a pro-metastatic mediator through tissue remodeling [22–24].

This review discusses the current understanding of how Hpa2 is regulated in heath and disease. We describe avenues for future research to further understand the role of Hpa2 in human health and disease. For a detailed discussion of the (patho)physiological roles of Hpa2, see the comprehensive review by Vlodavsky [25].

## Hpa2 isoform regulation and their subcellular localization

Four Hpa2 isoforms (Table 1) were annotated by expressed sequence tags, with isoform Hpa2c (592 aa) thought to be the most abundant [1]. The Hpa2c protein sequence shares the highest homology with Hpa1 (42%) and is exported to the cell surface via the secretory pathway. On the cell surface, Hpa2c binds peri- and extracellular HS [2]. Although all four isoforms contain the same signal peptide directing them into the endoplasmic reticulum (ER), earlier studies suggested that Hpa2a and Hpa2b remain within the cell interior [2]. Observations from our laboratory indicated that these two isoforms are also transported via the secretory pathway and are at least partially localized on the cell surface (unpublished observation). Another study detected three Hpa2 isoforms in human plasma samples [26]. A recent study detected nuclear Hpa2 in epithelial cells of healthy breast tissue and also showed, interestingly, that the ratio of nuclear Hpa2 to cytoplasmatic Hpa2 correlated inversely with breast cancer disease progression, raising the hypothesis that nuclear Hpa2 has protective effects in breast cancer [27]. However, the mechanisms through which Hpa2 is transported into the nucleus in breast tissue, and how this process is regulated, are unclear. The authors consider the possibility that, through the strong HS–Hpa2 binding affinity, nuclear HS pulls Hpa2 into the nucleus [27]. Possibly only a particular Hpa2 isoform shows nuclear enrichment. Further studies are needed to understand how expression of the different isoforms is regulated and how the isoforms differ in function and subcellular localization. Such studies will need to include analysis of the subcellular localization of each isoform, alone, followed by detailed comparative analyses. Additionally, isoform-specific antibodies would help map tissue distributions and subcellular localizations.

**Table 1:** Hpa2 isoforms

Isoform name	NCBI accession	Length (amino acids)
Hpa2a	NM_001166245.1	480
Hpa2b	NM_001166244.1	534
Hpa2c	NM_021828.5	592
Hpa2x	NM_001166246.1	548

## Regulation of Hpa2 during development

In general, Mendelian syndromes provide a clue to gene functions. UFS is a rare and potentially devastating autosomal-recessive disease. UFS comprises both incomplete urinary bladder emptying and a facial grimace upon smiling. The disease features mutations in *Hpa2*. Symptoms of UFS are already detectable in the second or third pregnancy trimester, when an enlarged bladder (megacystis) and dilated upper urinary tracts (hydronephrosis) appear in prenatal ultrasonography screens [7]. Accordingly, Hpa2 was detected in human tissue samples during later stages of the first trimester, when the detrusor muscle starts to differentiate and is innervated with autonomic nerves. At this timepoint, Hpa2 was immunodetected within nerve fascicles located between muscle bundles [28]. Concordantly, Hpa2 expression was detected in murine E14 bladder walls and pelvic ganglia innervating bladder smooth muscle tissue [11,29]. Interestingly, *Hpa2* expression remains postnatally high in murine [10,30] and human [8] bladder tissue and reaches relative levels that are 3-fold higher in adult compared with newborn mice [10]. Considering the strong Hpa2 expression in adult bladder tissue, the absence of a bladder phenotype in mice with conditional knockout of Hpa2 during adulthood is surprising [31]. In contrast, mice with constitutive knockout of Hpa2, generated via gene trap mutations, show megacystis and voiding dysfunction [10,11,30].

It is not known whether Hpa2 function in the fully developed bladder differs from that in the developing bladder, or whether loss of Hpa2 in adults is compensated for by another gene. Lopes et al. were able to alleviate the UFS-like phenotype in mice by using an adeno-associated virus gene therapy approach to deliver Hpa2 into neonatal mice [32] suggesting that the UFS phenotype is reversible after the initial bladder mis-innervation and opening potential avenues for therapeutic cure of the disease [32]. Studies in lower vertebrates affirm Hpa2 expression in peripheral neural tissue during development. Frogs, *Xenopus* embryos, express Hpa2 during early larval development in the developing neural tube and in the adult bladder [33]. Recently, we detected Hpa2 expression in dorsal root ganglia of 24-hour-old zebrafish embryos. This staining was temporally restricted, as it faded away when fish were examined for Hpa2 expression 24 hours later [6].

Taken together, the above results show that Hpa2 is specifically expressed in peripheral neuronal tissue across vertebrates, and that in mammals, Hpa2 is necessary for proper nerve patterning and progression for bladder innervation enabling synergistic bladder functioning. The necessity of Hpa2 for bladder function is well characterized. However, key questions remain to be investigated, including “What are the molecular mechanisms underlying Hpa2 function in axon guidance and nerve patterning?” and “Are the effects mediated by extra- or intracellular Hpa2? What is the function of Hpa2 in the adult bladder?”.

In addition to its role in peripheral nervous system development, Hpa2 may function in earlier developmental stages. This idea is supported by two studies that independently reported a failure to breed homozygous zebrafish [6] or mice [25] lacking Hpa2 gene function induced by CRISPR-Cas9 technology. We observed induction of Hpa2 expression during zebrafish gastrulation (unpublished observation), which may explain why zebrafish with homozygous loss of Hpa2 fail to develop further. It is possible that a similar Hpa2 expression pattern exists in mice, but answering this question will require experimental validation. In addition, the biological mechanisms underlying activation of *Hpa2* gene expression specifically during gastrulation in zebrafish, and ensuring proper development, require further study. Researchers have shown that Hpa2 gene trap mice are viable yet die a couple of weeks after birth, which is another intriguing phenomenon, as it cannot be attributed to the bladder phenotype or defects in early embryonic development [10,11]. Autopsies revealed that the mice showed no major organ abnormalities in lungs or kidneys [11]. Studies in *Xenopus* showed that Hpa2 is also expressed in the hindgut [33], and expression data from mice show *Hpa2* expression in the small and large intestine [11,33]. Possibly, the Hpa2 gene trap mice die at a young age because of defects in nutrient reabsorption in the gastrointestinal tract. Together, these studies point out the complexity of the networks regulating Hpa2 expression during different development stages and in different organs. Regulatory mechanisms common to different stages or organs remain to be uncovered.

## Hpa2 regulation under physiological conditions

Early studies showed that Hpa2 exhibits a highly regulated expression profile in different human tissues. *Hpa2* is expressed across brain tissue, throughout the intestine, in the bladder, uterus, prostate, and testis. Hpa2 is not expressed in heart, lungs, and kidneys [1,8]. The studies made no specifications about age and sex. Data in the Human Protein Atlas show a similar Hpa2 expression pattern, with pronounced expression in the brain, esophagus, urinary bladder, vagina, cervix, and small intestine [34]. Adolescent mice (postnatal day 30) show a partially similar Hpa2 expression profile, with expression most prominent in the bladder, followed by the intestine, but showing a virtual absence of Hpa2 expression in other tissues, including skeletal muscle, kidney, heart, and brain cortex [10]. Recent studies of Hpa2 protein localization show strong expression in the adult liver and pancreatic tissue of mouse and human tissue samples [6]. Zebrafish larvae and adults also showed pronounced hepatic expression [6,35]. In sum, in adult animals, Hpa2 shows pronounced expression in bladder tissue, is expressed in various other organs, and circulates in the bloodstream. The primary source of Hpa2 is not entirely clear; however, secretory organs such as mammary glands, pancreas, and liver may fuel the systemic vasculature with Hpa2.

## Hpa2 regulation under disease conditions

### Malignancies

Examination of Hpa2 expression in different human cancers including head and neck [2], gastric [13,14], pancreatic [18], hepatocellular [36], breast [15,27,37], bladder [16], cervical [36], and colon [17] cancer showed that, overall, decreased Hpa2 expression is associated with more severe tumor phenotypes and lower survival rates. One study examined the methylation status of the *Hpa2* promotor in colorectal carcinoma samples [17]. Hypermethylation is generally considered to repress gene transcription, a phenomenon frequently observed in tumors to dwarf the expression of tumor-suppressor genes [38]. The investigators found that hypermethylation of the *Hpa2* promotor is associated with poor patient prognosis and is mediated by the polycomb repressive complex 2 [17]. Mechanistically, Hpa2 overexpression in colorectal carcinoma cell lines up-regulated the tumor suppressors p21 and p53 and led to growth arrest of cancer cells in the G1 phase, as well as decreased tumor volumes in xenograft models [17]. A different regulatory mechanism for Hpa2 was found in breast cancer cells. miRNA-15b-5p represses Hpa2 by binding the 3′UTR region of the Hpa2 transcript, thereby leading to a more aggressive breast cancer phenotype characterized by increased proliferation and invasiveness [37]. Conditional Hpa2 knockout mice show a pancreatic phenotype characterized by decrease in weight of the pancreas and histological abnormalities. Moreover, Hpa2 deficiency was associated with a marked stimulation of pancreatic Hpa1 enzymatic activity indicating the inhibitory effect of Hpa2 on Hpa1 activity [31]. Closer analysis revealed widespread acinar-to-adipose tissue trans-differentiation in Hpa2-deficient pancreases. The reaction of Hpa2-deficient pancreatic tissue to cerulein-induced pancreatitis (a ten-amino-acid oligopeptide that stimulates smooth muscle and increases digestive secretions) was more pronounced than that of wild-type pancreatic tissue. This pronounced reaction resulted in an inflamed pancreas and, in combination with a carcinogen, led to development of neoplastic lesions, supporting the idea that Hpa2 acts as a tumor suppressor [31]. These reports align with findings that Hpa2 is expressed in pancreatic tissue of mice and human [6] and highlights the importance of Hpa2 in later stages of development. Together, these studies characterize Hpa2 as a tumor suppressor, with Hpa2 expression correlated positively with a patient’s prognosis.

### Kidney disease

Studies in different glomerular kidney disease conditions have shown that *Hpa2* gene expression and protein abundance change in response to disease. Renal cortical Hpa2 expression decreased in several experimental conditions in mice: experimental glomerulonephritis induced by lipopolysaccharide; antibody treatment directed against glomerular basement membrane proteins; streptozotocin-induced diabetic nephropathy in mice; and Adriamycin (doxorubicin)-induced nephropathy [39]. Interestingly, use of streptozotocin to induce diabetic nephropathy resulted in increased *Hpa2* expression if mice were on an Hpa1 knockout background [39]. However, the study did not present results for a phenotype in Hpa1-deficient mice and thus did not indicate whether or not the elevated *Hpa2* expression corresponds with a less severe streptozotocin-induced phenotype in Hpa1-deficient mice. It would be interesting to investigate whether *Hpa2* expression is impacted directly by Hpa1 or is affected by the disease progression. A study found that Hpa1 expression is not altered in conditional Hpa2 knockout mice versus wild-type mice [31]. On the other hand, Hpa1 and Hpa2 have been reported to be expressed in different tissues [1], and thus counter-regulation on a transcriptional level is possible. Analysis of plasma samples from patients in septic shock revealed lower abundance of both Hpa1 and Hpa2 compared with healthy controls [21]. However, circulating levels of Hpa2 were decreased more severely, shifting the Hpa1/Hpa2 ratio in the direction of Hpa1 [21]. A similar loss of plasma Hpa2 was observed in critically ill COVID-19 patients [20]. Together, these studies show an overall trend of Hpa2 down-regulation at the transcriptional level under different disease conditions. However, studies have not yet investigated whether common biological mechanisms regulate Hpa2 expression in different disease conditions.

## Hpa2 gene regulation under stress conditions

*Hpa2* gene expression shows strong differential regulation among different tissues and (patho)physiological conditions. Notably, *Hpa2* expression is up-regulated under different types of cellular stress. The cellular stress response is the wide range of molecular changes that take place in cells in response to environmental stressors, including temperature extremes, exposure to toxins, and mechanical damage. This phenomenon was convincingly demonstrated for *Hpa2* gene expression via *in vitro* studies of cells exposed to various stress conditions [36]. Thapsigargin, an inhibitor of the sarcoplasmic/ER calcium ATPase (SERCA), induced *Hpa2* gene expression in various malignant and non-malignant cell lines including pancreatic adenocarcinoma cell lines (Panc-01, Panc-02, AsPC1, CFPAC, Capan-1), HT1080 and HOS sarcoma cells, HeLa cervical cancer cells, HEK293T cells, and primary murine embryonic fibroblasts [18,36], suggesting a cell-type-independent mechanism regulating *Hpa2* expression on a transcriptional level. Notably, the effect of thapsigargin worked synergistically with that of hypoxic conditions (2% oxygen), as the two conditions together led to a remarkable increase in *Hpa2* gene expression ( > 20-fold) and protein abundance in Panc-01, HeLa, and HT1080 cells compared with the use of thapsigargin alone [36].

A similar pattern was observed for tunicamycin, an inhibitor of N-glycosylation and inducer of the unfolded protein response in eukaryotic cells [18,36]. Additionally, stressors including *cis*-platinum-induced cytotoxic stress, heat shock (42°C), and MG132-induced proteotoxic stress-induced *Hpa2* gene expression in HT1080 and HOS sarcoma cells lines, HEK293 cells, and primary murine fibroblasts [14,18,36]. The studies also show that this stress response includes up-regulation of protein kinase RNA-like ER kinase (PERK)-dependent genes including the genes for pro-apoptotic CHOP, the chaperone BiP, ATF4, and increased phosphorylation of eukaryotic initiation factor 2. PERK inhibition reduced *Hpa2* and *ATF4* gene expression [18,36]. Together, these observations established the model that *Hpa2* is a stress response gene that is up-regulated via the (PERK)-arm of the unfolded protein response pathway.

Providing additional evidence for a role of Hpa2 in the stress response, two putative binding sites for activating transcription factor 3 (ATF3) were characterized within the human *Hpa2* promotor region [36]. ATF3 is activated by a range of stress stimuli including metabolic and inflammatory signals and is an important regulator of endocrine organs such as liver and pancreas [40]. Luciferase reporter assays using the putative ATF3 binding sites of the *Hpa2* gene revealed induction of luciferase activity under thapsigargin and hypoxic conditions [36], as observed for *Hpa2* gene expression [18,36]. Importantly, fibroblasts isolated from ATF3 knockout mice failed to show induction of *Hpa2* gene expression upon thapsigargin and hypoxia treatment [36]. However, ATF3-deficient murine fibroblasts did show induction of *Hpa2* gene expression under proteotoxic stress conditions, suggesting that not all stress inducers activate *Hpa2* via ATF3 [36]. Thus, other regulatory elements of the Hpa2 promotor region await to be discovered and characterized. Notably, Hpa2 overexpression itself in pancreatic adenocarcinoma cell lines induced expression of the ER stress response proteins Bip, CHOP, and activating transcription factor 4 (ATF4) [18,36]. This finding led to the idea that *Hpa2* expression is induced by ER stress, with the ER stress then exacerbated by increased Hpa2 levels, thus creating a vicious positive feedback cycle [18]. This scenario may explain the anti-proliferative effects of Hpa2 in various tumor models including pancreatic, gastric, ovarian, and neck carcinoma and squamous sarcoma, where Hpa2 overexpression causes ER stress which in turn leads to growth arrest and apoptosis [18]. However, it is not known whether the effects of Hpa2 are mediated from the interior of the ER, or from the cell surface, where the vast majority of Hpa2c is expected to localize. Recent observations detected Hpa2 protein in hepatic and pancreatic tissue of zebrafish and mammals [6]. However, studies have not yet investigated whether ATF3 controls *Hpa2* expression in these endocrine organs or whether *Hpa2* gene expression is up-regulated in response to organ stress, leading to increased production of Hpa2 and thus increased plasma levels of Hpa2.

**Figure 2:**
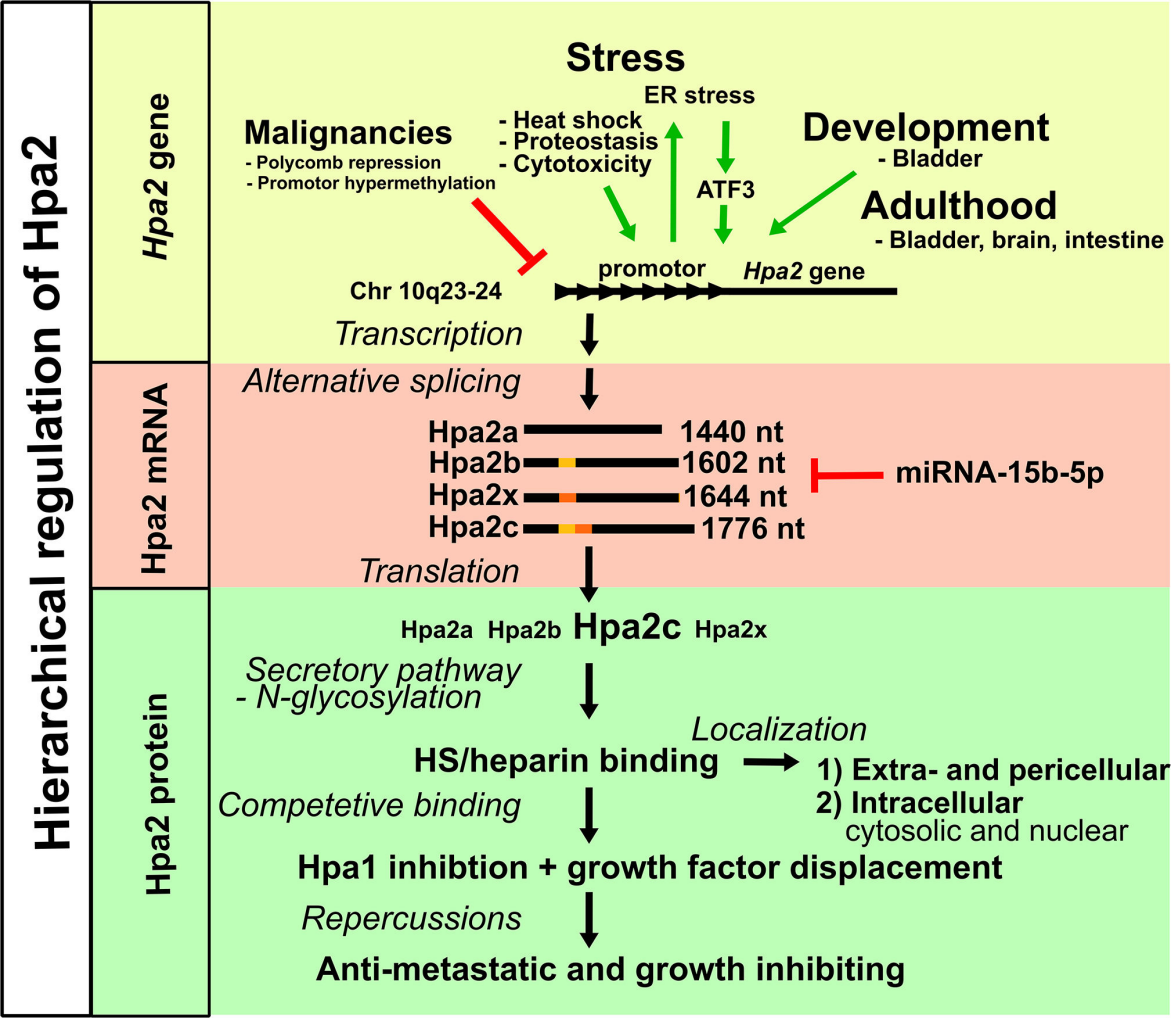
Hierarchical regulation of Hpa2. Hpa2 is regulated on the transcriptional level by promotor activation and repression. Alternative splicing produces four different transcripts of Hpa2, which are regulated by miRNA-15b-5p. The translated Hpa2 isoforms enter the secretory pathway. N-glycosylation can modulate protein function and localization. The Hpa2-HS interaction may further influence the localization of Hpa2. Hpa2 competes with Hpa1 and HS-binding growth factors for HS binding.

In summary, Hpa2 abundance is highly variable among different tissues, developmental stages, and disease conditions. Regulation is carried out on multiple levels, starting at the transcriptional level (Figure 2). Understanding these regulatory mechanisms for Hpa2 will help understand the diseases in which Hpa2 is implicated and will have potential to lead to novel treatment strategies to induce Hpa2 such that patients can benefit from its protective effects. Nonetheless, in particular, basic science knowledge is lacking. Hpa2 is a molecule that interferes with basic functions regulating extra- and pericellular matrices. Hpa1 splits a critical component of certain proteoglycans, namely, HS structures. That Hpa2 is unique and novel, and has many unexplained functions, is now known. Our laboratory is specifically interested in how Hpa2 regulates HS-dependent processes in the vasculature, which we believe is pivotal in numerous vascular diseases, including diabetic microangiopathy and sepsis. What is needed now is beyond description. We need structural data describing how HS proteoglycans, Hpa1, and Hpa2—the “non-heparanase” protein—interact at a molecular level.

**Perspective summary**

- Hpa2 is implicated in seemingly unrelated disease conditions including developmental, malignant, and cardiovascular diseases. Understanding the regulation and function of Hpa2 will contribute to further deciphering these complex diseases and pave the ways for novel treatment strategies.
- Hpa1 cleaves HS, while Hpa2 does not, although both bind HS. Hpa2 is highly regulated on the expression level and plays different functional roles during ontogenesis and pathogenesis. Hpa2 is associated with beneficial effects for human health, and Hpa2 is primarily down-regulated under pathological conditions.
- We require a better understanding of Hpa2 regulation in terms of isoform regulation, subcellular localization, promotor activation, and repression as well as protein degradation and posttranslational modification.

## Open Access

This article has been published open access under our Subscribe to Open programme, made possible through the support of our subscribing institutions, learn more here: https://portlandpress.com/pages/open_access_options_and_prices#conditional

## Abbreviations

ATF3: activating transcription factor 3
ATF4: activating transcription factor 4
ER: endoplasmic reticulum
GAGs: glycosaminoglycans
HS: heparan sulfate
Hpa1: heparanase 1
Hpa2: heparanase 2
PERK: protein kinase RNA-like ER kinase
SERCA: sarcoplasmic/ER calcium ATPase
UFS: urofacial syndrome
